# The Expanding Therapeutic Perspective of CCR5 Blockade

**DOI:** 10.3389/fimmu.2017.01981

**Published:** 2018-01-12

**Authors:** Luca Vangelista, Sandro Vento

**Affiliations:** ^1^Department of Biomedical Sciences, Nazarbayev University School of Medicine, Astana, Kazakhstan; ^2^Department of Medicine, Nazarbayev University School of Medicine and University Medical Center, Astana, Kazakhstan

**Keywords:** CCR5, CCL5, inflammation, HIV-1, cancer

## Abstract

CCR5 and its interaction with chemokine ligands have been crucial for understanding and tackling HIV-1 entry into target cells. However, over time, CCR5 has witnessed an impressive transition from being considered rather unimportant in physiology and pathology to becoming central in a growing number of pathophysiological conditions. It now turns out that the massive efforts devoted to combat HIV-1 entry by interfering with CCR5, and the subsequent production of chemokine ligand variants, small chemical compounds, and other molecular entities and strategies, may set the therapeutic standards for a wealth of different pathologies. Expressed on various cell types, CCR5 plays a vital role in the inflammatory response by directing cells to sites of inflammation. Aside HIV-1, CCR5 has been implicated in other infectious diseases and non-infectious diseases such as cancer, atherosclerosis, and inflammatory bowel disease. Individuals carrying the CCR5Δ32 mutation live a normal life and are warranted a natural barrier to HIV-1 infection. Therefore, CCR5 antagonism and gene-edited knockout of the receptor gained growing interest for the therapeutic role that CCR5 blockade may play in the attenuation of the severity or progression of numerous diseases.

## Introduction

From its discovery, CCR5 has been a key player in HIV-1 entry into target cells and, together with its chemokine ligands, helped in understanding and tackling HIV-1 infection (1, 2). CCR5 predominates among the chemokine co-receptors used by HIV-1 for cell entry, and R5-tropic HIV-1 strains are those most commonly transmitted in the early stages of infection. A 32 base pair deletion within the CCR5 gene leads to a non-functional gene product that does not reach the cell surface, and subjects with a homozygous CCR5Δ32 deletion are protected from HIV-1 infection (3).

The discovery and implication of CCR5, CXCR4, and their chemokine ligands in HIV-1 pathogenesis triggered massive research efforts that cross-fertilized many biomedical fields related to the chemokine system and regulation. In recent years, evidence has accumulated that CCR5 and its ligands may play a role in various inflammatory diseases, as cellular activation of CCR5 normally happens through chemokine binding, which then regulate intracellular trafficking and protective cellular and humoral responses. Indeed, the migration of lymphocytes to inflammatory areas is controlled by chemokine gradients (4). CCR5 may also be relevant in the development of various types of cancer, as tumor cells directly secrete or induce fibroblasts to secrete CCL5, which maintain proliferation of CCR5-positive cancer cells. Finally, CCR5 may play a role in autoimmune diseases such as rheumatoid arthritis and multiple sclerosis (MS).

In this mini-review, we describe several aspects related to the pathophysiology of CCR5, discuss its possible dispensability, and analyze its blockade as a comprehensive therapeutic perspective.

## Is CCR5 A Discardable Troublesome Receptor?

**Figure 1:**
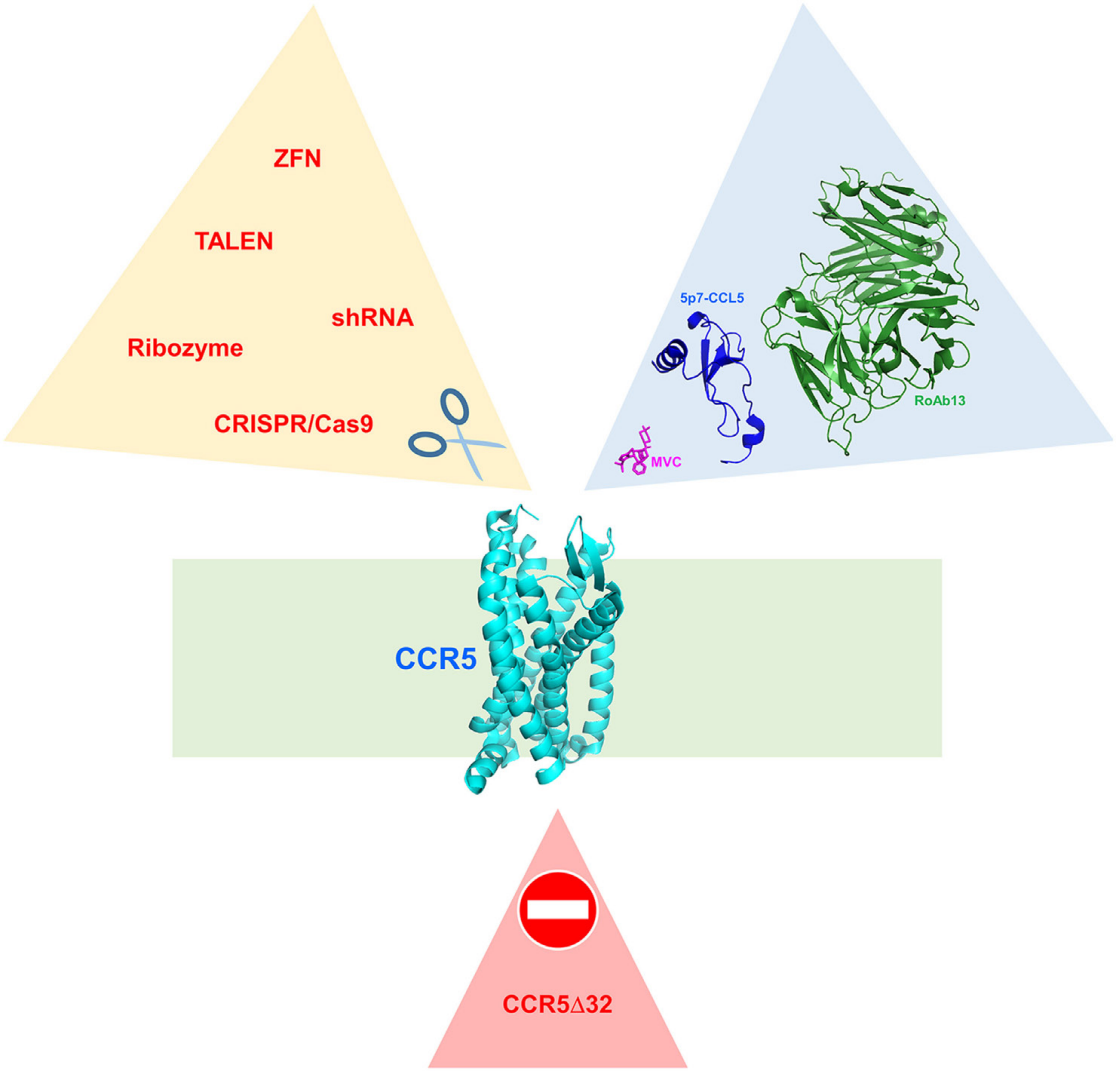
CCR5 blockade. CCR5 blockade may occur (i) naturally (triangle on the bottom), by the CCR5Δ32 deletion that prevents the receptor to be transported to the cell surface; (ii) *via* gene editing strategies (triangle on the left) that ablate the CCR5 gene; or (iii) by receptor antagonism (triangle on the right) using different molecular entities. The cell membrane is represented with a green rectangle. Ribbon representation of CCR5 and 5p7-CCL5 three-dimensional structures were generated using PyMOL from PDB entry 5UIW (10), MVC from PDB entry 4MBS (11), and the FAB fragment of RoAb13 from PDB entry 4S2S (12).

Soon after its discovery and implication for HIV-1 entry, CCR5 has been the subject of extensive research as a possible new player in the search for preventative and therapeutic solutions to the HIV-1 infection pandemic (2). A radical approach to CCR5 targeting could be the elimination of the receptor by gene editing, in an attempt to resemble the naturally occurring Δ32 deletion (5) (Figure 1). This approach has its foundation on the fact that individuals homozygous for the CCR5Δ32 deletion are seemingly healthy. The proof of concept for CCR5 elimination has been provided by the so-called Berlin patient, an HIV-1 infected person who, after a double CCR5Δ32 stem cell transplant, has remained HIV free (6, 7). However, the CCR5Δ32 mutation dates thousands of years and individuals that carry it naturally may have adapted their chemokine system to physiologically balance the absence of a functional CCR5 (8). Therefore, the effect of CCR5Δ32 stem cell transplants and artificially induced CCR5 knockouts should be considered carefully, and individuals subjected to these treatments should be followed up for a long time (9). Similar caution needs to be taken when acting on CCR5 with more conventional approaches (e.g., using CCR5 antagonists), although drug discontinuation is likely to restore normal CCR5 expression and function.

Therefore, CCR5 blockade is still an open question, as well as the genetic mechanism and environmental pressure that generated the CCR5Δ32 mutation. While HIV-1 cannot be accounted for the origins of the CCR5Δ32 mutation, these have been initially attributed to selective pressure by pathogens such as *Yersinia pestis* or variola virus. However, these hypotheses have been dismissed in favor of an older selection event connected to a different pathogen (13). Indeed, the CCR5Δ32 gene has been detected in Bronze Age skeletons (14) and is estimated to have emerged ~5,000 years ago, predating the time during which smallpox and plague became widespread human pathogens (13).

## CCR5 in Pathology

A role for CCR5 has been suggested in numerous diseases, many involving the nervous system. CCR5 ligands are produced in the central nervous system (CNS) by microglia, astrocytes, endothelial cells, and even neurons (15, 16). The cerebrospinal fluid (CSF) of patients with relapsing-remitting MS has CCR2^+^CCR5^+^ T_H_1 cells during a relapse; CCR5^+^CD8^+^ T cells and CCR5^+^ monocytes are higher in the CSF than in the blood of patients with the disease, and CCR5 is expressed in inflammatory cells infiltrating the CNS *in vivo* (17, 18). CCR5 is also expressed on immune cells within inflammatory lesions in MS and may contribute to recruitment of these cells to the inflamed tissue or to their activation. Finally, the expression of CCR5 ligands has been shown at sites of inflammation in MS (19). Interestingly, MS can develop in people who are homozygous for the CCR5Δ32 mutation. The CCR5Δ32 allele is not associated with MS risk (20, 21), but the disease seems to be less severe in carriers of the allele (22), suggesting that CCR5 antagonists might diminish disease activity.

In contrast, homozygosity for the CCR5Δ32 allele is overrepresented in patients with symptomatic West Nile virus infection (23, 24) and is associated with severe meningoencephalitis in tick-borne encephalitis virus infections (25). Most likely, CCR5 facilitates clearance of these infections by promoting leukocyte trafficking to the CNS, a proof of its beneficial effects for human health (23). CCR5 may instead be detrimental in patients with cerebral malaria, in brain samples of whom it was found to be upregulated (26). The CCR5Δ32 allele seems to be associated with resistance to Crimean-Congo hemorrhagic fever (CCHF) virus infection, at least in the Turkish population (27). Indeed, CCL3, CCL4, and CCL5, natural ligands of CCR5, are associated with CCHF, and their levels are increased in adult patients with the infection (28).

In an emerging infectious disease, dengue virus infection, an association has been found with CCR5 expression, and the infection induces the expression of CCR5 ligands (29).

In its pathogenesis, *Toxoplasma gondii* produces a chemokine mimic that triggers CCR5, a subtle mechanism likely used to warrant *T. gondii* survival in the host (30). However, in the absence of CCR5, mice succumb to infection with uncontrolled parasite growth, altered lipid metabolism, hepatic steatosis, and widespread intestinal damage with ileum necrosis and prominent neutrophils infiltrate (31). Whether CCR5 is essential for *T. gondii* infection control in humans is unknown.

Poxviruses use chemokine receptors, including CCR5, to infect target cells; however, their molecular mechanism of receptor usage is distinct from that of HIV-1 (32). In a mouse model based on intranasal vaccinia virus infection, CCR5 expression in T cells contributes to the dissemination of the virus to the lungs and beyond; the data suggest that the role of CCR5 in vaccinia virus infection is not redundant and that CCR5 may be necessary for systemic infection *in vivo* (33).

*Staphylococcus aureus* is the cause of a large number of deadly infections worldwide, and the emergence of antibiotic-resistant *S. aureus* strains represents a steadily increasing global threat. The bicomponent pore-forming leukotoxin ED (LukED) is used by *S. aureus* to compromise the host immune system and cause deadly infectivity, and the gene for LukED is present in numerous clinically relevant *S. aureus* strains (34). LukE binds to human (and mouse) CCR5 on T cells, macrophages, and dendritic cells (35); subsequently, a bicomponent octamer formed by alternate LukE and LukD monomers assembles on the surface of target cells. The pores formed by LukED ultimately lead to cell death. LukED kills CCR5^+^ cells *in vivo* in mice, and animals lacking CCR5 are protected from mortality due to *S. aureus* infection (35). Even though both LukE and gp120 target CCR5, they use different determinants on the receptor (36). Interestingly, CCR5 antagonism by maraviroc (a small chemical HIV-1 entry inhibitor) confers mice with resistance to lethal *S. aureus* infection. Maraviroc completely blocks LukED pore formation *in vitro* and therefore toxicity toward CCR5^+^ cells (35). Therefore, the use of CCR5 antagonists to counteract *S. aureus* infection is an interesting example of antibacterial intervention, alternative or even complementary to antibiotics. In light of the debate on the emergence of the CCR5Δ32 mutation, the deadly effects of *S. aureus* infections on humankind and LukE tropism for CCR5 might have generated the ancient selection of the CCR5Δ32 allele (35).

CCR5 may also have a role in autoimmune diseases. In rheumatoid arthritis, increased levels of CCR5 ligands CCL3, CCL4, and CCL5 are found in the synovial fluid (37, 38), and the CCR5Δ32 variant seems to protect from the disease (39). However, maraviroc does not efficiently control inflammation in this setting (40).

CCR5 appears to be relevant in atherosclerosis and the development of related diseases (41). A meta-analysis of 13 studies assessed whether individuals carrying the CCR5Δ32 variant could be either protected or at risk for atherosclerosis-related cardiovascular diseases and indicated that the CCR5 Δ32-positive genotype (Δ32/Δ32 or wt/Δ32) increases the risk of atherosclerotic disease only in Asian populations (42). In a recent report, CCR5 has been described as a non-redundant, essential receptor for the homing of CD4 T cells that exacerbate atherosclerosis (43).

An increased expression of CCL5 has been detected as early as 8 days *postpartum* in a mouse model of tubulointerstitial kidney disease, an inflammatory disorder that causes progressive kidney damage and renal failure (44). It might be possible that CCL5 participates in the early cascade of event bridging the unfolded protein response (caused by an uromodulin mutation) to inflammation, although further investigations are needed (44).

CCL5 expression is increased in inflammatory bowel disease (IBD), likely pointing to a contribution by CCL5 in the progressive tissue destruction during the inflammatory processes (45). A recent investigation provided evidence that blocking CCR5 either by genetic ablation or by pharmacological inhibition with maraviroc rescued mice from colitis in both acute and chronic models (46). The latter is particularly interesting since the live microbicide strategy developed to provide vaginal *in vivo* delivery of CCL5-based HIV-1 entry inhibitors by engineered lactobacilli (47) could indeed be applied in the context of IBD, where lactobacilli are naturally resident commensal bacteria.

CCR5 has been implicated in the development of various types of cancer, including breast cancer, ovarian and cervical cancer, prostate cancer, colon cancer, melanoma, Hodgkin lymphoma, and multiple myeloma (48). Cancer cells secrete CCL5 or induce fibroblasts to secrete CCL5, which sustain the proliferation of CCR5-positive tumor cells (48); recruit T-regulatory cells and monocytes with suppressive functions; cause osteoclast activation; and favor bone metastasis, neo-angiogenesis, and dissemination of cancer cells to distant organs (49). CCL5 has been reported to provide antitumor adjuvanticity or, conversely, to promote carcinogenesis, depending on the tumor environment (50). These opposite effects appear to be justified by the type of cancer, CCR5 expression by cancer cells, and localization of CCL5 expression. Hence, CCR5 antagonism or activation may be circumstantially tailored to provide an antitumor effect (50–53).

Finally, a multivariate analysis of unrelated HLA-matched bone marrow transplantation for hematologic malignancies conducted in Japan showed that the recipient CCR5-2086A/A genotype was significantly associated with a lower relapse rate, resulting in better disease-free and overall survival rates than other variations (54). Therefore, the recipient CCR5-2086A/A genotype affects the induction of the graft-versus-tumor effect without augmenting the development of graft-versus-host-disease (GVHD), and CCR5 genotyping in transplant recipients may be useful in determining pretransplantation risks.

In a recently published comparison of a cohort of patients enrolled in a trial of reduced-intensity allo-hematopoietic stem cell transplantation with standard GVHD prophylaxis plus maraviroc and a contemporary control cohort receiving standard GVHD prophylaxis alone, maraviroc treatment was associated with a lower incidence of acute GVHD without increased risk of disease relapse and with reduced levels of gut-specific markers (55). Maraviroc treatment increased CCR5 expression on T cells and reduced T cell activation in peripheral blood without increasing the risk of infections. These data suggest that maraviroc protects against GVHD through modulation of allo-reactive donor T cell responses.

## CCR5 Gene Editing

As discussed earlier, CCR5 knockout induced by gene therapy techniques is a strategy to reproduce the naturally occurring CCR5Δ32 deletion (56) (Figure 1). However, CCR5 abrogation by gene editing has been so far considered exclusively for the cure of HIV-1 (5). Zinc finger nucleases (57) have been used on CCR5 (58) and recently reviewed for their therapeutic potential and clinical trial implications (59). Other CCR5-targeted gene editing techniques include the CRISPR/Cas9 nuclease system and the transcription activator-like effector nuclease (60), as well as short hairpin RNAs (61) and ribozymes (62).

## CCR5 Antagonists

In 2007, maraviroc, a negative allosteric modulator of the CCR5 receptor and therefore competitive CCR5 inhibitor, was approved for clinical use as an HIV-1 entry inhibitor that showed additional efficiency in antiretroviral-pretreated patients (63). Thus, maraviroc is far the single success story emerged from the massive pharmaceutical effort spent in the development of small chemical compounds acting as chemokine antagonists (64); many hurdles were associated with the lack of receptor specificity and the toxicity derived from it. The effect of CCR5 antagonism by maraviroc in HIV-1-infected individuals has been reported to lead to transient early treatment increase in the CD4 count and a late treatment increase in the CD8 count, which may imply a recovery of the cell-mediated immunity (65). Overall, maraviroc treatment did not seem to interfere with normal homeostasis, rather to improve it (66, 67), and ameliorate inflammatory processes in HIV-1 and beyond (68).

In the effort to attain HIV-1 entry inhibition by CCR5 blockade, CCR5 must be engaged by antagonist ligands, to avoid sustained receptor activation that could generate unwanted pro-inflammatory conditions. As described above, the participation of CCR5 in a large array of chronic inflammatory diseases makes CCR5 antagonism (or, more drastically, gene-edited CCR5 knock out) an elective therapeutic option.

Two other CCR5 antagonists have been evaluated in clinical trials in HIV-infected individuals and have failed to progress. In phase II trials in treatment-naive patients of vicriviroc, a noncompetitive allosteric CCR5 antagonist (69, 70), viral rebound with continued treatment was observed (71), and in treatment-experienced patients, there was an increase in malignancies (72). Aplaviroc, a spirodiketopiperazine derivative, caused severe hepatotoxicity in infected patients in phase II clinical trials (73).

Cenicriviroc is a relatively new CCR5 antagonist presently assessed in clinical trials; it inhibits both CCR2 and CCR5 receptors and has good oral absorption (74). Cenicriviroc may offer other benefits in addition to its anti-HIV activity and is also currently in clinical trials testing its ability to reduce fibrosis in patients with non-alcoholic steatohepatitis and primary sclerosing cholangitis (75).

CCL3, CCL4, and CCL5, natural agonist ligands of CCR5, represent obvious templates for the development of protein-based CCR5 antagonists. However, CCR5 activation by these chemokines required them to be molecularly switched into antagonists. A long-lasting success story is represented by the CCL5 derivatives saga (76). Populated by several different approaches targeting the chemokine *N*-terminus, it ultimately led to highly potent variants that interact with CCR5 as antagonists (77) and are ~200-fold more potent than maraviroc in blocking HIV-1 *in vitro*.

Another protein-based approach to CCR5 antagonism is the development of monoclonal antibodies (mAbs) against CCR5 (78). PRO 140, a humanized IgG4 mAb derived from the murine mAb PA14 (79, 80), is currently in a phase III clinical trial (81). PRO 140 efficiently inhibits HIV-1 gp120 binding to CCR5 and, with lower potency, chemokines interaction with the receptor (78). Another promising anti-CCR5 mAb is CCR5mAb004, a fully human IgG4 also being tested in clinical trials (82). RoAb13 is also capable of blocking HIV-1 infection (83), and the three-dimensional structure of its Fab has been recently solved (12). Interestingly, naturally occurring anti-CCR5 antibodies have been suggested to contribute to the maintenance of homeostasis (84).

Blockade of CCR5 with antagonists is increasingly adopted to counteract inflammatory diseases and infections where this receptor plays a relevant role. Being FDA approved and a small chemical compound, maraviroc is the CCR5 antagonist of election; however, protein-based CCR5 antagonists could be equally or even more effective. The three-dimensional structure of the complex between CCR5 and maraviroc (11) helped significantly in understanding GPCR conformational modularity and visualized the deep insertion of maraviroc in the CCR5 ligand cavity. Small chemical compounds have a relatively lower production cost and might be easier to administer, compared to protein drugs. However, last generation CCL5-based antagonists (77) provided *in vitro* anti-HIV-1 potency far superior than that of maraviroc and grant a virtually absent development of HIV-1-resistant strains (85), which is not the case for maraviroc. In a recent breakthrough in structural biology (10), the three-dimensional structure of the complex between CCR5 and 5p7-CCL5 (a potent CCR5 antagonist) (77) has been solved, revealing the extensive and deep area of CCR5 occupancy by 5p7-CCL5. This intimate molecular interaction largely justifies the impossibility for HIV-1 to generate escape mutants since gp120 occupies a similar cavity on CCR5 [modeled in Ref. (10)]; also the virus cannot generate a gp120 molecule able to circumvent the presence of 5p7-CCL5 or similar CCL5 variants. Ultimately, the high CCR5 affinity of these CCL5 variants could be exploited in the different pathological conditions where CCR5 plays a potentially crucial role.

## Conclusion and Perspectives

Biomedical investigations are elucidating a growing role played by CCR5 in several inflammatory diseases, and a number of microorganisms hijack CCR5 to exert their tropism. In this scenario, CCR5 blockade is conceived as a relatively harmless therapeutic option (Figure 1). This option is implemented either by biochemical blockade of the receptor using CCR5 antagonists or by excision of the receptor by gene editing strategies. Which of the two strategies is preferable may depend on the disease dynamics and the actual CCR5 dispensability suggested by the CCR5Δ32 allele present in individuals living a seemingly healthy life.

## Author Contributions

Both authors have made a substantial, direct, and intellectual contribution to the work and approved it for publication.

## Conflict of Interest Statement

The authors declare the absence of any commercial or financial relationships that could be construed as a potential conflict of interest.
